# A retrospectively registered pilot randomized controlled trial of postbiotic administration during antibiotic treatment increases microbiome diversity and enriches health-associated taxa

**DOI:** 10.1128/iai.00390-25

**Published:** 2025-11-28

**Authors:** Jonas Schluter, William Jogia, Fanny Matheis, Wataru Ebina, Alexis P. Sullivan, Kelly Gordon, Elbert Fanega de la Cruz, Mary E. Victory-Hays, Mary Joan Heinly, Catherine S. Diefenbach, Un Jung Kang, Jonathan U. Peled, Kevin R. Foster, Aubrey Levitt, Eric McLaughlin

**Affiliations:** 1 Institute for Systems Genetics, New York University Grossman School of Medicine 12296, New York, New York, USA; 2 Department of Microbiology, New York University Grossman School of Medicine 12296, New York, New York, USA; 3 Laura and Isaac Perlmutter Cancer Center, New York University Grossman School of Medicine 12296, New York, New York, USA; 4 Postbiotics Plus Research, Houston, Texas, USA; 5 Patients Emergency Room & Hospital 650486 , Baytown, Texas, USA; 6 Department of Neurology, New York University School of Medicine 12296 , New York, New York, USA; 7 Adult Bone Marrow Transplantation Service, Department of Medicine, Memorial Sloan Kettering Cancer Center 691931, New York, New York, USA; 8 Weill Cornell Medical College 12295 , New York, New York, USA; 9 Department of Biology, University of Oxford 98459, Oxford, United Kingdom; 10 Department of Biochemistry, University of Oxford 98957, Oxford, United Kingdom; University of California San Diego School of Medicine, La Jolla, California, USA

**Keywords:** microbiome injury, postbiotic

## Abstract

Antibiotic-induced microbiome injury, defined as a reduction of ecological diversity and obligate anaerobe taxa, is associated with negative health outcomes in hospitalized patients, and healthy individuals who received antibiotics in the past are at higher risk for autoimmune diseases. Postbiotics contain mixtures of bacterial fermentation metabolites and bacterial cell wall components that have the potential to modulate microbial communities. Yet, it is unknown if a fermentation-derived postbiotic can reduce antibiotic-induced microbiome injury. Here, we present the results from a single-center, randomized placebo-controlled trial involving 32 patients who received an oral, fermentation-derived postbiotic alongside oral antibiotic and probiotic therapy for non-gastrointestinal (GI) infections. At the end of the antibiotic course, patients receiving the postbiotic (*n* = 16) had significantly higher fecal bacterial alpha diversity (+40%, inverse Simpson index) compared to the placebo group (*n* = 16), and the treatment was well-tolerated. Analysis of 157 longitudinal fecal samples revealed that this increased diversity was driven by enrichment of health-associated taxa, notably obligate anaerobic Firmicutes, particularly Lachnospiraceae. In contrast, *Escherichia/Shigella* species, often linked to pathogenicity and antibiotic resistance, were reduced in postbiotic-treated patients at the end of antibiotic treatment and remained lower up to 10 days later. Our findings suggest that postbiotic co-administration during antibiotic therapy may augment health-associated gut microbiome composition and mitigate antibiotic-induced microbiome injury.

Trial registration ISRCTN30327931 retrospectively registered.

## INTRODUCTION

Antibiotic therapy is the most effective treatment to fight bacterial infections and indispensable in modern medicine; it can cure mild disease and save lives in severe cases of bacteremia (1), organ invasion, and sepsis (2). Recently, however, evidence has accumulated that oral antibiotic treatment collaterally injures the human microbiome (3–11). Antibiotics may interfere with commensal bacterial growth, or can kill health-associated bacterial populations (3, 9), a phenomenon that may have been vastly underestimated (3). Such disruption of the commensal microbial ecosystem can persist for months following antibiotic exposure (7) and is seen in otherwise healthy individuals (12) as well as severely immunocompromised, hospitalized patients who, in some instances, lose most of the normally resident bacteria (8–11). Clearance of the normal flora can enable persistent colonization by pathogens, e.g., *Clostridioides difficile*, cause recurrent, difficult-to-cure, infections (13, 14), and may delay immune reconstitution in chemotherapeutically treated cancer patients (15).

Antibiotic-induced interference with bacterial reproduction and killing of gut bacterial populations lead to shifts in the gut microbiome ecosystem that can be characterized by a decline in alpha diversity (9, 10, 16). Alpha diversity metrics are summary measures quantifying the ecological composition of a microbiome sample; they may capture the number of taxonomic groups (e.g., more taxa increase diversity) and their abundance distributions (e.g., more evenly distributed taxon abundances increase diversity) (17). Therefore, they have been used to quantify microbiome injury at the ecosystem level (18). Notably, antibiotic use and loss of bacterial microbiome alpha diversity have been associated with health risks, including higher mortality in hematopoietic stem cell transplantation (19, 20), translocation of microorganisms from the gut into the bloodstream (often resulting in bacteremia and sepsis) in immunocompromised patient populations (10, 11, 16, 21, 22), increased risk for inflammatory bowel disease (23), and severity of autoimmune and allergic diseases (24–28). Furthermore, loss of bacterial diversity can affect cross-kingdom interactions: depletion of bacterial competitors may allow for fungal species to expand (29), and fungal expansions were associated with increased risk of fungal bloodstream infections in cancer patients (16) as well as fungal urogenital infections in otherwise healthy women (30). Recently, antibiotic exposure has been associated with worse response to cancer immunotherapies (31–35), and direct links between perturbation of the microbiome and human immune system modulation are emerging (15, 36, 37).

A diverse microbiome, predominantly populated by obligate anaerobe taxa, is, thus, associated with good health. As a result, means to restore the microbiome following antibiotic exposure are much sought after. Autologous fecal microbiota transplantation (auto-FMT) can recover antibiotic-depleted bacteria using fecal materials harvested and stored prior to treatment (38). Heterologous FMTs with material from healthy donors (allo-FMT) have shown promising results in improving inflammatory bowel disease (39), response to immune therapy (40), and successfully reduced *C. difficile* abundances and recurrence (41, 42). FMTs, however, come with risks (43): antimicrobial-resistant strains are prevalent in the microbiome (11) and can sometimes cause life-threatening complications during FMT (44). Furthermore, matching donor to recipients for optimal engraftment remains an unsolved challenge (45). Nevertheless, recently, the U.S. Food and Drug Administration has approved two FMT-based therapies: a rectally administered single dose of prepared stool from healthy donors and a fecal microbiota-based oral treatment, both to prevent the recurrence of *C. difficile* infection (46, 47). In addition to large community transfers via FMT, administration of selected live microbial species, termed probiotics, has been established, albeit with conflicting data on efficacy (48–50), and variable recipient colonization (51, 52). While probiotics are generally assumed to be safe, their use has been associated with adverse outcomes (48), such as bacteremia (53), fungemia (54), and bowel ischemia (49) especially in immunocompromised and critically ill patients. Facilitation of probiotic colonization following antibiotic-induced depletion of recipient commensals may also lead to persistent dysbiosis and delay recovery of the pretreatment microbiome (55). Modulating resident commensal microbial ecology without the addition of new microbial strains and species offers alternative strategies toward safe and robust microbe-targeted therapies, for example, through the use of prebiotics. These are substances intended to provide nutrients to specific, desired microbial populations and have recently been shown to affect microbiome communities (37). Surprisingly, however, a stronger beneficial, anti-inflammatory effect was observed resulting from increased consumption of fermented foods (37). Thus, products of fermentation may offer a novel therapeutic avenue to manipulate the microbiome.

As such, an emerging alternative to live therapeutics, which we explore here, is the use of postbiotics. Postbiotics is an umbrella term that comprises complex mixtures of metabolites produced by bacteria, for example, during fermentation, as well as cell wall components and other dead cell components (as well as residual viable cells) (56).

Emerging pre-clinical and clinical studies indicate health-beneficial effects of postbiotics (37, 57, 58). Thus, postbiotics may offer a safe novel therapeutic avenue to target the microbiome. In particular, we hypothesize that reduction of antibiotic-induced microbiome damage could prevent antibiotic-induced impairment of novel immunotherapies and health sequelae (31, 59). However, it is not yet known if postbiotics can reduce microbiome diversity loss during antibiotic treatment. Here, we describe the results of a retrospectively registered single-center randomized controlled trial to assess the ability of a fermentation-derived postbiotic to prevent antibiotic-induced injury to the commensal microbiome in patients receiving oral antibiotics. Following a course of antibiotics, we collected stool samples at five timepoints and profiled the composition of bacterial populations. Our data indicate that treatment with a postbiotic during a course of oral antibiotics supports gut microbial ecosystem stability and health-associated taxa.

## RESULTS

### Overview of study design and intervention

**Fig 1:**
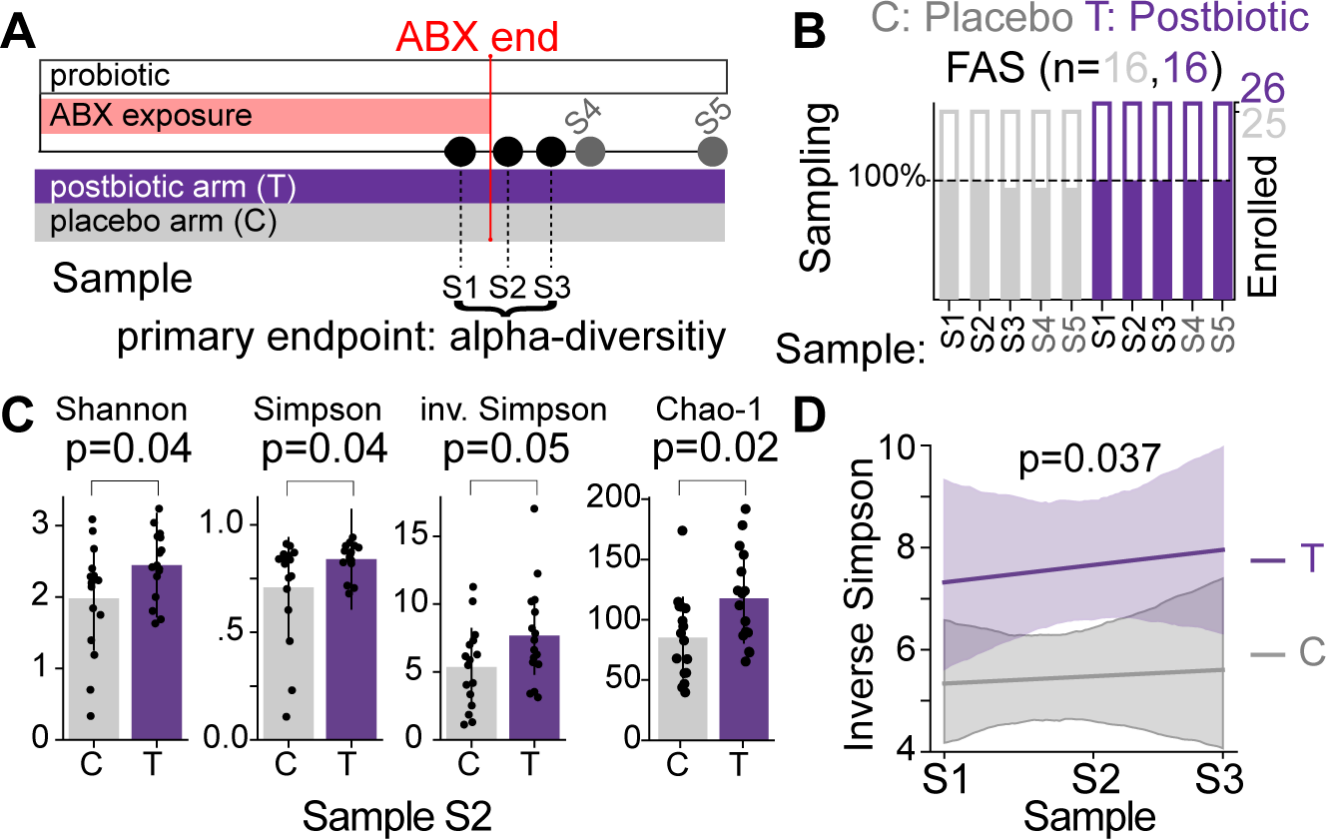
Postbiotics significantly increased bacterial alpha diversity at the end of an antibiotic course. (**A**) Sample collection protocol and study design; primary endpoint of the trial was alpha-diversity (inverse Simpson index) during the end of the antibiotic course (S1–S3), and additional endpoints were alpha-diversity (inverse Simpson) at later time points (S4, S5).(**B**) Enrollment and sample collection efficiency in percent of planned vs obtained; Full analysis set, (FAS): 100% = 16 samples per arm; cohort report in Fig. S1. (**C**) Alpha diversity directly after finishing the antibiotic course (S2, *P*-values from two-sided *t*-tests). (**D**) Inverse Simpson alpha diversity across timepoints S1–S3 was significantly higher in treated (purple) than control (gray) patients, see also Fig. S2; *P*-value from linear mixed effects model (38). Significance of differences in IVS was calculated using a mixed effects model for repeated measures with time treated as a categorical variable (38); significance between arms at individual timepoints was determined using two-sided *t*-tests.

A single-center, randomized placebo-controlled trial of postbiotic treatment (T, *n* = 16) vs placebo (C, *n* = 16) was conducted to measure bacterial alpha diversity as a primary endpoint at the end and directly after finishing a course of oral antibiotics. The goal was to determine feasibility, safety, and a preliminary efficacy assessment of the procedure for prevention of medication-induced injury of the gut microbiota (Study title: Randomized controlled trial of PBPGP22 to affect microbiome composition; https://www.isrctn.com; trial registration ISRCTN30327931). Otherwise healthy ambulatory adult patients (Table 1) presenting to a single clinical site in Houston, Texas, between 05 and 06/2018 for non-gastrointestinal infections were recruited. After giving informed consent, patients were provided with take-home stool sample collection kits, a supply of a commercially available probiotic, and a supply of the placebo (control, C) or postbiotic (treatment, T) to be taken during and 10 days beyond the antibiotic course (administered antibiotics are listed in Table 2) along with the commercial probiotic and their standard-therapy antibiotic (Fig. 1; Fig. S1, CONSORT report [60]). We chose to include the probiotic background, which included strains of *Lactobacillus* and *Bifidobacterium* because it was the standard recommendation by the participating physicians when administering antibiotics. Moreover, by including an existing treatment in our study, our design represented a relatively stringent test of the ability of a postbiotic to further improve outcomes. A total of 157 stool samples were collected longitudinally, for up to five timepoints (S1–S5) relative to the end of a full course of prescribed antibiotics per patient (Fig. 1A).

**TABLE 1:** Patient characteristics and indications by treatment arm, with statistical tests for *P* value calculations indicated

Characteristic		Control	Treatment	*P* value
Age		39, (20, 60)	45, (20, 64)	0.95 (*t*-test)
Sex	Female	10	8	0.72 (Chi2)
	Male	6	8	
Disease				
	Sinusitis	5	3	
	Cellulitis	2	1	
	Kidney infection/UTI	1	0	
	Abscess	1	0	
	Ear infection	2	0	
	Cyst	1	0	
	Pharyngitis/Strep	1	6	
	Bronchitis	1	2	
	Laceration	0	1	
	Toothache	0	1	
	Other	2	2	

**TABLE 2:** Antibiotic exposures by treatment arm

Treatment	Antibiotic	N
T	Amoxicillin/Clavulanic acid	5
	Amoxicillin	2
	Amoxicillin/Clavulanic acid & Cefalexin	1
	Azithromycin	1
	Cefalexin	1
	Cefalexin/Trimethoprim/Sulfamethoxazole	1
	Ciprofloxacin	1
	Ciprofloxacin/Metronidazole	1
	Clindamycin	1
	Levofloxacin	1
	Penicillin	1
C	Amoxicillin/Clavulanic acid	7
	Amoxicillin	2
	Ciproflaxin/Metronidazole	1
	Ciprofloxacin	1
	Ciprofloxacin	1
	Clindamycin	1
	Trimethoprim/Sulfamethoxazole/Cefalexin	1
	Azithromycin	1
	Amoxicillin/Clavulanic acid & Clindamycin	1

It was hypothesized that microbiome injury would be maximal at the end of a completed course of antibiotics. Therefore, the primary endpoint was set as bacterial alpha diversity, measured by the inverse Simpson index on three timepoints at the end of the antibiotic course: on the day prior to finishing the antibiotic treatment (S1), directly after completing the antibiotic (S2) and 1 day thereafter (S3). Among patients who entered the study, stool sampling rates were high, with only a single sample from S3 missing in the final analysis set (FAS, target participant number: 25, FAS: 32, Fig. 1B). Patients were given a phone number to call to report any side effects and were contacted via text messages prior to fecal sample collection; there were no grade three or higher side effects reported (Table S1).

### Bacterial alpha diversity at the end of an antibiotic course is high in patients receiving the postbiotic

Microbiome profiling by amplification and sequencing of the V4 region of the 16S rRNA gene was performed on DNA collected from 157 samples (Table S2). Bacterial alpha diversity measured at the end of the antibiotic treatment (S2) was significantly higher in postbiotic-treated patients compared with placebo recipients for different alpha diversity metrics, capturing evenness and richness (Fig. 1C, Shannon: +24%, *P* = 0.046; Simpson: +19%, *P* = 0.048; inverse Simpson: +43%, *P* = 0.056, +39% Chao-1, *P* = 0.02, *t*-test). Time series analysis (38) of longitudinally collected samples (Fig. S2) showed that postbiotic-treated patients had significantly higher alpha diversity during the end phase of their antibiotic treatment (Fig. 1D, *P* = 0.037, linear mixed effects model). Together, these results indicate that the addition of this postbiotic to an orally administered antibiotic course is clinically feasible, well-tolerated, and significantly increases gut microbial alpha diversity.

### Postbiotic treatment is associated with characteristic bacterial signatures

**Fig 2:**
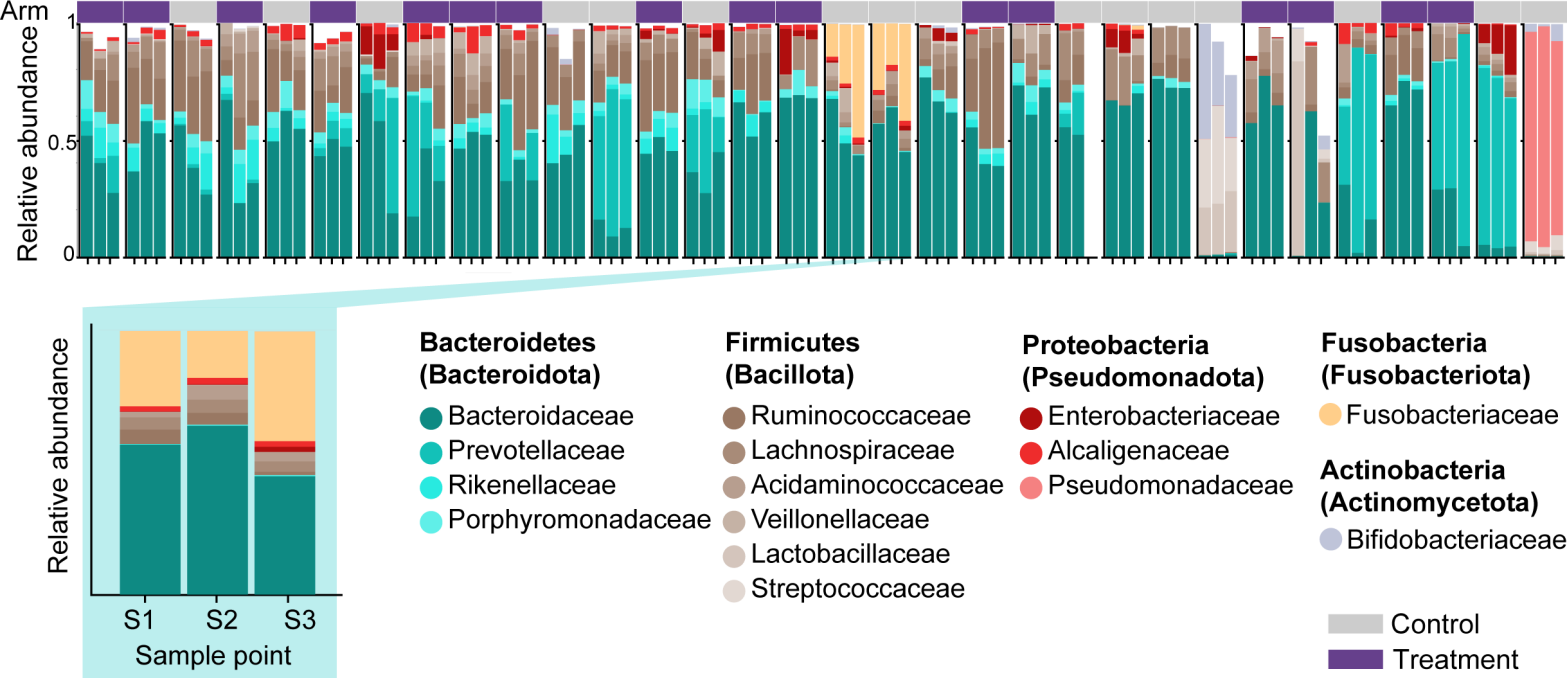
Bacterial compositions in longitudinally collected fecal samples. Bacterial family compositions in samples S1–S3 for each patient; families of identical higher taxonomic groups in similar colors, one patient time course enlarged. Patients are sorted by average alpha diversity across samples; treatment arm (gray box: control, purple box: treatment) is indicated above the time courses.

**Fig 3:**
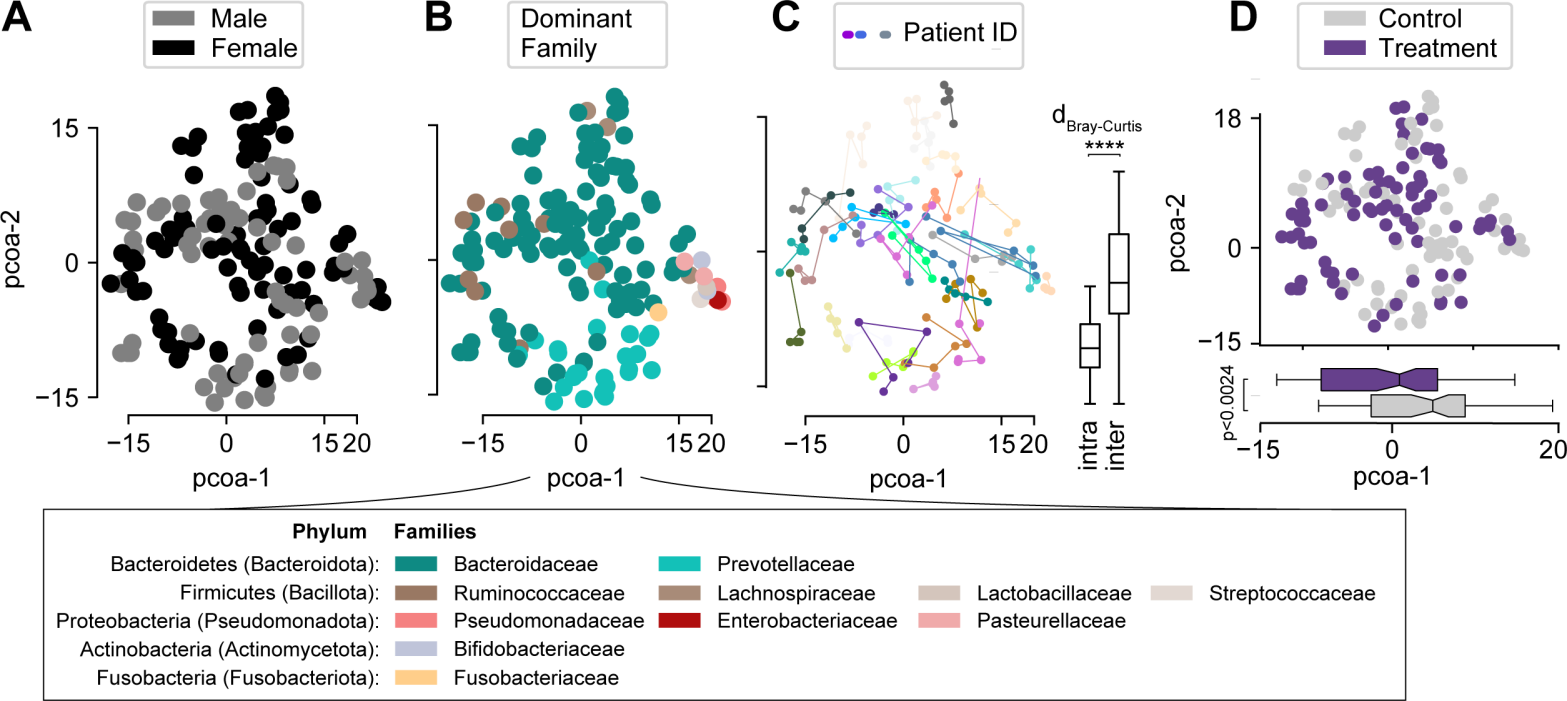
Beta diversity analysis of bacterial compositions. (**A–D**) PCoA plots of bacterial relative abundances in all 157 samples, colored by sex (**A**), most abundant bacterial family per sample (**B**). (**C**) Patients’ compositional trajectories visualized on the PCoA plot, with boxplot showing intra- and inter-subject Bray-Curtis dissimilarities. (**D**) PCoA labeled by treatment, with significant differences between the groups along the first principal coordinate indicated (****: *P* < 10^−4^, two-sided *t*-test). Differences in sample-by-sample distances were calculated with the adonis2 function from the vegan library for the R programming language.

We next sought to analyze differences between microbial ecosystems in treated vs control patients at a higher resolution than resolved by alpha diversity metrics. For this, we first visualized the individual patients’ compositional time courses by plotting the relative abundances of the most abundant bacterial families (Fig. 2), which indicated compositional variation across patients and changes over time in several individuals. To analyze compositional variability, we next performed a principal coordinate analysis (PCoA) on sample-by-sample Bray-Curtis dissimilarity calculated on operational taxonomic units’ (OTUs) relative abundances (Fig. 3). Samples from patients of either sex showed no significant differences directly after finishing antibiotics (S2, *P* = 0.368, PERMANOVA) and spanned the observed range of all bacterial compositions (Fig. 3A, *P* = 0.57, GLMMMirKAT) (61). In most samples, obligate anaerobe Bacteroidetes taxa represented the most abundant bacterial families (Fig. 3B), whereas samples from both experimental arms contained only low relative abundances of the genera corresponding to the probiotics taken by all patients (median relative genus abundance:*Bifidobacterium* [C: 3.9e−4, T:4.3e−4], *Lactobacillus* [C: 1e−4, T: 0.9e−4]; Fig. S3). Consecutive samples from the same patient tended to localize near one another, with significantly lower intra- than inter-patient variability during the transition from antibiotics into the post-antibiotic period (Fig. 3C), even though the observed microbiome dynamics in several patients spanned wide regions of the compositional space. While samples from treated and control patients localized across the PCoA space, a statistical analysis indicated that the samples from treated patients differed consistently in their composition from those observed in control patient samples (Fig. 3D), which suggested characteristic gut bacterial communities were enriched in postbiotic treated patients.

### Postbiotic treatment is associated with an enrichment in health-associated taxa and a reduction in disease-associated taxa

To analyze which taxa were consistently higher in treated patients or lower alongside the reduced diversity in control patients, we compared the relative abundance profiles between the two patient groups directly after finishing the antibiotic course (S2), and over time (S1–S3). To account for the compositional nature of 16S relative abundance data, we calculated the centered-log ratio (CLR) transformation of relative taxon abundances across all samples. To reduce sparsity of the OTU-level data, we aggregated relative abundances at the phylum, family, or genus levels by summing the corresponding OTU abundances prior to CLR transformation.

**Fig 4:**
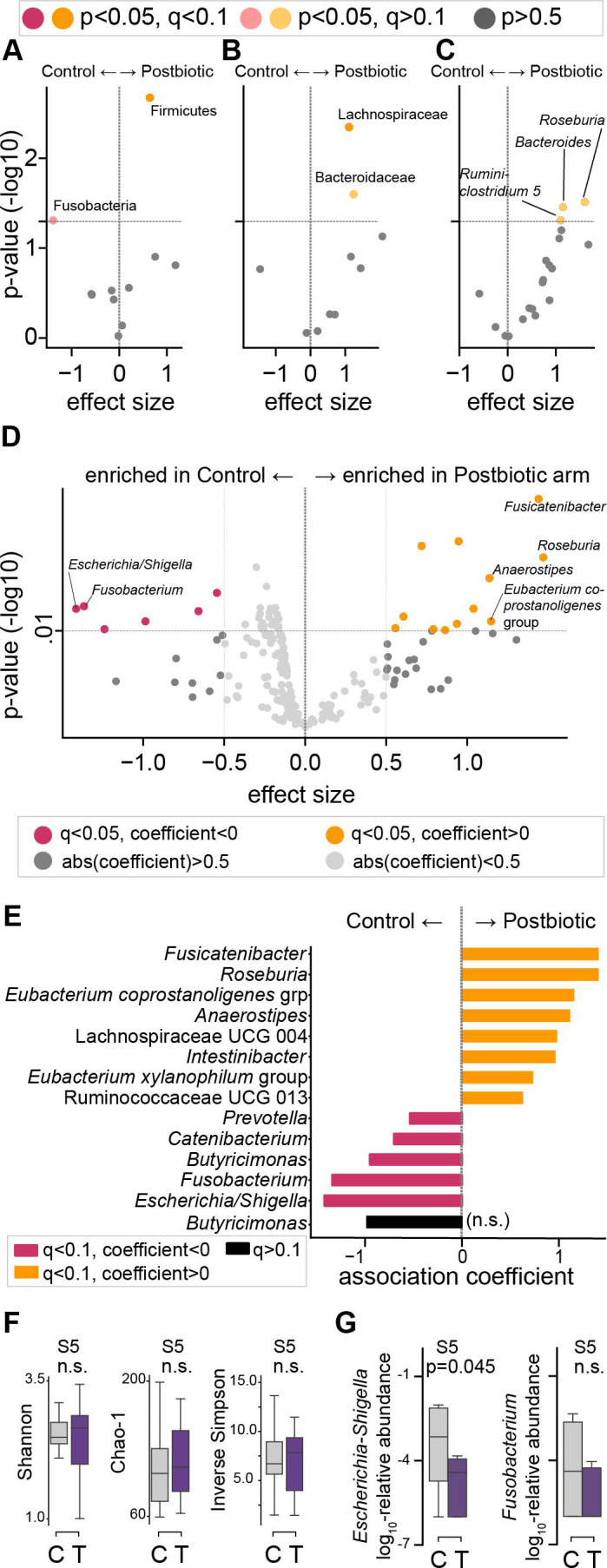
Health-associated taxa are enriched in postbiotics treated patients. (**A–C**) Volcano plots of association coefficient effect sizes between CLR-transformed taxon abundances and treatment arm; control (left), treatment (right) in samples collected at the end of an antibiotic course (S2). Negative log_10_-transformed *P*-values of association coefficients from 10 most abundant phyla (**A**), families (**B**), and 20 most abundant genera (**C**); significant associations in color (**A–C**, light color: univariate *P*-value < 0.05, full color: *q*-value < 0.05, 0.1 FDR, Benjamini/Hochberg method). (**D**) Volcano plot of associations between CLR-transformed relative genus abundances with treatment or control in samples S1–S3 (100 most abundant genera analyzed). (**E**) Significant genera from D re-analyzed with a mixed effects model using random intercepts per patient to account for multiple samples per patient; colored bars indicate significant association coefficients (pink: higher in control, yellow: higher in treatment arm) after multiple hypothesis correction via the Benjamini/Hochberg method (FDR: 0.1, *q* < 0.05). (**F**) Alpha diversity at sample point 5 by treatment arm. (**G**) Select disease associated genus abundances at sample point 5 by treatment arm (F, G: two-sided *t*-test). Single time points were analyzed with univariate linear models using the *ols* function of the statsmodels package, multiple hypothesis correction was performed with the Benjamini/Hochberg procedure using the fdrcorrection function of the statsmodels.stats.multitest library for the Python programming language. The multivariate analysis of significant hits from the univariate screen was performed with the LogisticRegressionCV class from the scikit-learn package for the Python programming language. n.s.: non-significant.

Statistical comparison of the CLR-transformed phylum relative abundances directly after finishing the antibiotic course (S2) revealed a significantly higher abundance of Firmicutes among postbiotic-treated patients (Fig. 4A, *P*-value = 0.002, *q*-value < 0.05, 0.1 FDR). At higher taxonomic resolutions, we analyzed the 10 most abundant bacterial families and 20 most abundant genera after CLR-transformation: after correcting for multiple hypotheses, the family of Lachnospiraceae was significantly enriched in treated patients (univariate *P*-value = 0.004, *q* < 0.05, 0.1 FDR). Bacteroidaceae (family-level analysis), and *Roseburia*, *Bacteroides*, and *Ruminiclostridium* 5 (genus-level analysis) were enriched in treated patients at S2 (Fig. 4B and C), but the individual significance of univariate analyses did not remain significant after correcting for multiple hypotheses.

To investigate if these compositional differences were consistent over time, we analyzed CLR-transformed genus abundances longitudinally in samples S1 to S3, i.e., in time points directly surrounding the end of the antibiotic course, as well as at later time points. Using a more stringent false discovery rate (0.05 FDR) as well as a minimal effect size cutoff (0.5), this revealed several genera with higher abundances in treated patients, including *Anaerostipes*, *Eubacterium coprostanoligenes* group, *Fusicantenibacter*, and *Roseburia*, and genera with lower abundances in treated patients, including *Fusobacterium* and *Escherichia/Shigella* (Fig. 4D; Fig. S4). We then tested the identified 13 differentially abundant genera with a mixed effects model that accounted for repeated measurements (Fig. 4E), which confirmed the associations found in the univariate pooled analysis screen (Fig. 4D). While CLR transformation can reduce some compositional data limitations, we also sought to confirm the taxon associations observed in univariate analyses in combined analyses of the entire composition. Consistent with the univariate results, we found similar associations when performing multivariate analyses using regularized regression approaches (Fig. S5), which confirmed a trend toward enrichment of commensal obligate anaerobe genera among treated patients.

We and others have previously found that antibiotics can deplete anaerobe taxa associated with immune modulation during cancer therapies (15, 32, 33). Though not a predefined trial end point, we next performed targeted analyses to compare abundances of a selected set of such taxa; we found higher abundances of Lachnospiraceae, which comprise *Faecalibacterium*, Ruminococcaceae, which comprise *Ruminococcus*, and Verrucomicrobiaceae, which comprise *Akkermansia*, in treated patients (Fig. S6).

Interestingly, enrichment of health-associated anaerobe taxa and concurrent lower levels of facultative anaerobe, pathobiont-comprising taxa in treated patients appeared to persist beyond the immediate end of the antibiotic exposure (Fig. S7) even though alpha diversity in control patients had already approached similar levels to treated patients 10 days after the antibiotic course ended (Fig. 4F and G; Fig. S8). In particular, facultative-anaerobe gamma-proteobacteria of the *Escherichia/Shigella* genus were reduced in treated patients even 10 days after finishing their antibiotic course (Fig. 4G; Fig. S7B). Taken together, these results support an enrichment of a health-associated microbial community in patients treated with a postbiotic during their antibiotic course.

## DISCUSSION

We here present results from a single-center randomized placebo-controlled trial to assess the effect of a postbiotic adjuvant on microbiome diversity during antibiotic therapy. Collateral injury to the resident gut microbiota caused by antibiotics has been described as a loss of bacterial ecosystem diversity (19), loss of commensal microbial taxa (9, 16), and increase of pathobionts, which can comprise antibiotic-resistant species (11). The enrolled patients were ambulatory and treated with oral antibiotics for non-gastrointestinal infections. Thus, the patient cohort studied here was at a lower risk for microbiome injury-induced complications than for example hospitalized blood cancer patients (11, 16, 19). In such high-risk patients, mortality is statistically associated with antibiotic-induced loss of diversity (19), and response to chimeric antigen receptor T cell therapy is negatively associated with antibiotic exposure (31, 35, 59). In hospitalized COVID-19 patients, another high-risk patient group, a combination of antibiotics and viral infection, was found to predispose patients to gut born bacteremia (10, 62). And even in otherwise healthy individuals such as those studied here, post-antibiotic complications such as diarrhea (63), post-antibiotic *Clostridioides difficile* infection (64), or vaginal candidiasis (65) are common, and selection for antimicrobial- resistant strains (AMR) in the gut microbiome may pose a potential health risk during future therapies. Therefore, reducing the negative collateral impact of antibiotics on the microbiome constitutes a critical unmet need.

Our results show that microbiome injury, assessed via multiple parameters, is attenuated when an oral postbiotic is co-administered with antibiotics: bacterial alpha diversity was significantly higher at the end of the antibiotic course in patients who received the postbiotic instead of the placebo, and this increased diversity was driven by commensal taxa. Health-associated obligate anaerobe organisms were higher in treated patients: *Fusicantenibacter*, which is depleted in rheumatoid arthritis patients (66), short-chain fatty acid-producing *Roseburia*, one of the most abundant genera in the healthy gut microbiome (67), the butyrogenic *Anaerostipes* genus, which comprises lactate-consuming species (68), and the *Eubacterium coprostanoligenes* group, which has been hypothesized to be involved in beneficial fat metabolism by reducing cholesterol (69). Conversely, *Escherichia/Shigella*, a genus of gram-negative facultative anaerobe species that comprise multiple antibiotic-resistant strains and pathobionts that can cause secondary bacteremia when translocating across a weakened gut barrier (10, 11), were significantly lower in treated than in control patients. Interestingly, we also observed that *Fusobacterium*, an obligate anaerobe taxon, was significantly lower in treated patients. Its predominant species, *F. nucleatum*, is commonly found in the oral microbiome and associated with the development of colorectal cancer when abundant in the gut (70, 71). It is plausible that the higher diversity of commensal gut bacteria in our treated patients improved colonization resistance and reduced gut colonization of *F. nucleatum* when swallowed. Furthermore, association studies found *Fusicantenibacter*, *Roseburia*, and *Anaerostipes*, which were enriched in postbiotic treated patients, to be the most consistently depleted taxa in Parkinson’s patients (72, 73), a disease that often manifests first in the gut. Lastly, the higher diversity of commensal organisms in treated patients may have suppressed opportunistic pathobiont expansion of *Escherichia/Shigella*, which was observed in control patients, likely through competitive exclusion (11, 74–76).

Limitations of this study include the variable types and duration of antibiotics, and consequently different duration of postbiotic treatments, and the administration of a commercial probiotic to both groups; however, the low abundances of probiotic-derived taxa suggest negligible influence on observed diversity differences between treatment arms. Probiotics were administered because when the study was designed, it was considered a potentially beneficial intervention. However, while some studies support this potential benefit (50), others have indicated that probiotics could hamper post-antibiotic microbiome recovery (55). Furthermore, interventions with high viable cell counts (e.g., probiotic or other live therapeutics) may harbor risks for immunocompromised patients (77, 78), who could benefit from reduced microbiome injury (19). Thus, future studies are warranted to validate the efficacy of the oral postbiotic outside the context of concurrent probiotic administration.

No baseline stool sample prior to antibiotics was collected due to the enrollment at an urgent care clinic where patients were instructed to start their treatment immediately. However, we speculate that the impact of antibiotics on the microbiome would be maximal at the end of an antibiotic course (7, 79), and that thus the increase in diversity observed among treated patients could reflect protection of the commensal microbiome. This is a plausible scenario because of the enrichment of commensal taxa in the microbiome of patients treated with the postbiotic, which did not include these taxa. The study results, while significant and of considerable magnitude, are based on a small cohort and a direct impact on patients’ health was not assessed in this trial, which focused on microbiome diversity as a primary outcome. Additional studies are needed to validate the herein described beneficial impact of postbiotic treatment on the microbiome and assess if the stabilization of the microbiome translates into a patient health benefit.

Supportive evidence for such potential benefits comes from the study of fermented plant-based foods, which were found to have a beneficial effect on the microbiome as well as the immune system (37). The postbiotic under investigation is a complex biologic product derived from the fermentation of medicinal plants by generally regarded as safe (GRAS) bacterial species (Table 3). Unlike fermented foods, the manufacturing of postbiotics generally includes a dedicated inactivation step that reduces live cell counts (56), and therefore, they are assumed to have a generally favorable safety profile over live therapeutic-based approaches. For the manufacturing of the postbiotic used in this trial, a fermentation process by specific species of *Lactobacillus* and *Bifidobacterium* was used, representing major taxa commonly present during the assembly of the infant microbiome (80). While the mechanism of action of the observed protection of gut microbiome diversity is not fully understood, we hypothesize that bacterial metabolites of these species, their products of secondary metabolism and signaling molecules may support species of the commensal adult microbiome, which have evolved to ecologically succeed early colonizers of the gut microbiome (81), increasing their resilience to external perturbations (82).

**TABLE 3:** Postbiotic composition

Component	Quantity in a capsule	Function
Ashwagandha root (*Withania somnifera*) extract	139.75 mg	Active ingredient: Co-fermented herb with *Lactobacillus* and *Bifidobacterium*
Elderberry fruit (*Sambucus nigra*) powder	143 mg	Active ingredient: Co-fermented herb with *Lactobacillus* and *Bifidobacterium*
Astragalus root (*Astragalus membranaceus*)	139.75 mg	Active Ingredient: Co-fermented herb with *Lactobacillus* and *Bifidobacterium*
Red lentils	227.5 mg	Bulking agent, nitrogen source

In conclusion, here we have presented evidence that co-administering a fermentation-derived postbiotic can increase gut microbiome diversity during antibiotic treatments. The increased diversity was associated with higher abundances of health-associated bacterial taxa, while genera comprising pathobionts were lower among postbiotic treated patients.

## MATERIALS AND METHODS

### Human subjects

Table 1 reports metadata, including age and sex, corresponding to the 32 healthy adult subjects enrolled in a single site double-blind randomized controlled study (trial registration ID: ISRCTN30327931). This study was reviewed and approved by New England Institutional Review Board, an independent IRB located in Needham, MA. All subjects were provided with an explanation of the study and gave informed consent in writing prior to the start of their participation in the study.** **

### Human subject study methods

Fifty-one ambulatory adult subjects were initially evaluated for eligibility (see inclusion and exclusion criteria used to evaluate subject suitability listed below), one was not eligible. Fifty of the subjects were then enrolled in a single site double-blind interventional study with a randomized-controlled design to test the hypothesis that patients receiving an oral postbiotic would present with higher bacterial alpha diversity, measured by the inverse Simpson index, during three time points at the end of an antibiotic course than patients receiving a placebo control. Analyzed timepoints were the final day of antibiotics, the first day after completing the antibiotics course, and the second day after completing the antibiotics course. Analysis was performed by time series analysis that accounts for repeated measurements and uses time as categorical predictors, as done before (38, 83). This study was reviewed and approved by New England Institutional Review Board, an independent IRB located in Needham, MA. Eligibility for enrollment was determined by the following inclusion and exclusion criteria:

Inclusion criteria

- Otherwise healthy adults with a body mass index (BMI) (18–28 kg/m²)
- Patients are prescribed a course of antibiotics with a duration of at least 5 days
- Patients have not had another course of antibiotics in the past 6 months
- Patients are willing to cease taking any other supplements

Exclusion criteria

- Patients suffering from gut related illness (regular and severe constipation, diarrhea, inflammatory bowel disorder, or inflammatory bowel disease).
- Patients who usually experience severe diarrhea (e.g., for more than 3 days) following antibiotic treatment.
- Patients who have a compromised immune system are taking any immune modulators or have an autoimmune disorder.
- Patients who are diabetic or take any blood pressure medication.
- Patients who have any known allergies to the probiotics (Table S3), or the herbs and bacteria used in the postbiotic formula (Table 3).
- Patients who are pregnant or nursing.

The double-blind randomization scheme for this study was as follows. Patients were given an unmarked box (the study kit) containing stool sample materials, and either control or treatment materials neither of which were marked. To blind study coordinators, boxes had been shuffled after assembly of all study kits, prior to the study. Kits contained barcodes that allowed identification of participants’ treatment arm after the participant handed over their samples. One of the subjects evaluated for participation was ineligible due to not meeting the inclusion criteria. Fifty subjects received the study kits. Seventeen subjects withdrew consent during follow-up; 6 and 12 subjects from the control and the treatment arm, respectively, without explanation. One patient from the treatment arm was removed from the study due to a second antibiotic prescription which was not declared during enrollment.

Treatment regimens were as follows. In their study kit, the control arm received a commercially available probiotic (Table 3) in unmarked capsules and a resistant starch placebo in an unmarked capsule; the treatment arm received the same commercially available probiotic in unmarked capsules and the fermentation-derived postbiotic in unmarked capsules. Subjects were instructed to take one capsule of each every day during their antibiotic course and for 10 days after finishing the antibiotic course.

Patients were given a phone number to call to report any side effects; no such phone calls were received. Prior to the collection of each stool sample, study coordinators were checking in with patients via text message to remind them of the sample collection and ask for side effects, collectively presented in Table S1. Patients were contacted on study days 1, 2, 3, 4, and 10, and there were no grade 3 or higher side effects reported.

### Eligibility criteria, ethical approval, and consent to participate

This study protocol was approved by the institutional review board (IRB) number: 120180088, trial registration ID: ISRCTN30327931. Otherwise healthy ambulatory adult patients (Table 1) presenting to a clinic in Houston, Texas, between 05 and 06/2018 for non-gastrointestinal infections were recruited. Participants were recruited by physicians. Onsite nurses, trained in human subject research, reviewed the consent form with the potential participants and answered any questions about the study and its potential risks. Informed consent was obtained from all participants. Participants were compensated with a $15 Amazon gift card per sample collected at the end of the study. If a participant did not complete the study, they were not penalized and rewarded for the samples they collected.

### Sample collection

After informed consent was obtained, participants were provided with 5 Diversigen OmniGene Gut kits for stool collection at home, a kit which stabilized samples at room temperature for up to 60 days. Patients were handed a work sheet listing their expected samples, recorded the date of sample collection, and shipped all samples in one batch. Samples were received and processed by a specialized contract research organization, Diversigen, (Houston, Texas).

### DNA extraction and bacterial 16S rRNA gene sequencing

DNA from human samples was extracted with PowerSoil Pro (Qiagen) on the QiaCube HT (Qiagen), using Powerbead Pro (Qiagen) plates with 0.5 mm and 0.1 mm ceramic beads. Human samples were prepared with a protocol derived from reference 44, using KAPA HiFi Polymerase to amplify the V4 region of the 16S rRNA gene. All PCR products were analyzed with the Agilent TapeStation for quality control and then pooled equimolar and sequenced directly in the Illumina MiSeq platform using the 2 × 250 bp protocol and the MiSeq Reagent Kitv2. Sample processing and sequencing were performed by Diversigen.

### Bioinformatic processing and taxonomic assignment

OTU tables were generated by Diversigen bioinformatics pipelines (detailed bioinformatic tools and settings in Supplementary Methods). Briefly, Useach v7.0 was used to process raw sequencing reads, and taxonomy was assigned via the Silva database v128.

### Statistical analyses

Unless otherwise stated, statistical analyses were performed with Python 3, version 3.10.4 and R, version 4.2.

We calculated the inverse Simpson (IVS) index from relative ASV abundances (*p*) with *N* ASVs in a given sample, $IVS=\;\frac{1}{\sum_{i}^{N}p_{i}^{2}}$. Regression analyses were conducted using the statsmodels package for the Python programming language, using the functions *ols* and *mixedlm* for ordinary least squares and mixed effects modeling, respectively. Relevant code of primary analyses and corresponding data tables are available in the supplementary materials.

To account for compositionality of the relative abundance data, we performed a centered log-ratio (CLR) transformation at different taxonomic aggregation levels, i.e., separate CLR for genus, family, and phylum abundances. Regression models were implemented using the statsmodels (using functions *ols* and *mixedlm*) (84) and penalized multiple variate regression models using the sklearn packages (using the function *LogisticRegression*) (85) for the Python programming language, and using the *nlme* (86) and *vegan* (87) library in R.

*P* values were adjusted as appropriate. A *P*-value < 0.05 was considered significant after adjustment for multiple comparisons. Unless otherwise stated, significance values are noted as follows: *P < 0.05, **P < 0.01, ***P < 0.001.

## Data Availability

Data, statistical test results, and relevant code to reproduce main analyses are available as supplementary materials. The 16S rRNA gene sequencing results are available as supplementary data files (Table S2). Raw sequencing reads are available on NCBI SRA, BioProject PRJNA1032616.

## SUPPLEMENTAL MATERIAL

Supplemental material iai.00390-25-s0001.docx Tables S1, S3; Fig. S1 to S8.

Table S2 iai.00390-25-s0002.xlsx Data and statistical model output.

ASM does not own the copyrights to Supplemental Material that may be linked to, or accessed through, an article. The authors have granted ASM a non-exclusive, world-wide license to publish the Supplemental Material files. Please contact the corresponding author directly for reuse.
