# Supplementary material for: A retrospectively registered pilot randomized controlled trial of postbiotic administration during antibiotic treatment increases microbiome diversity and enriches health-associated taxa
Source: Infect Immun. 2025 Nov 28;94(1):e00390-25. doi: 10.1128/iai.00390-25 (PMC12797998; doi:10.1128/iai.00390-25)
Supplement: Supplemental material — Tables S1, S3; Fig. S1 to S8. [file iai.00390-25-s0001.docx]

**A retrospectively registered pilot randomized controlled trial of postbiotic administration during antibiotic treatment increases microbiome diversity and enriches health associated taxa.**

Jonas Schluter^1-4*^, William Jogia^1,2^, Fanny Matheis^1^, Wataru Ebina^1,3^, Alexis P. Sullivan^1^, Kelly Gordon^4^, Elbert Fanega de la Cruz^5^, Mary E Victory-Hays^5^, Mary Joan Heinly^5^, Catherine S. Diefenbach^3^, Un Jung Kang^6^, Jonathan U. Peled^4,7,8^, Kevin R. Foster^4,9,10^, Aubrey Levitt^4^, Eric McLaughlin^4,5^

^1^ Institute for Systems Genetics, New York University Grossman School of Medicine, New York, NY, USA

^2^ Department of Microbiology, New York University Grossman School of Medicine, New York, NY, USA

^3^ Laura and Isaac Perlmutter Cancer Center, New York University Grossman School of Medicine, New York, NY, USA

^4^ Postbiotics Plus Research, Houston, TX, USA

## ^5^ Patients Emergency Room & Hospital 10133 I-10, Baytown, TX, USA

^6^ Department of Neurology, New York University School of Medicine, New York, NY, USA

^7^ Adult Bone Marrow Transplantation Service, Department of Medicine, Memorial Sloan Kettering Cancer Center, New York, NY, USA

^8^ Weill Cornell Medical College, New York, NY, USA

^9^ Department of Biology, University of Oxford; Oxford, UK

^10^ Department of Biochemistry, University of Oxford; Oxford, UK

***) correspondence: jonas.schluter@nyulangone.org**

- Postbiotic administration during antibiotic treatment increases bacterial alpha diversity
- Characteristic bacterial signatures are associated with postbiotic administration
- Health-associated taxa are enriched and disease-associated taxa are reduced by postbiotic treatment

**Supplementary Material**

**Table S1: Reported adverse events in three subjects of the control arm.**

|  | **Adverse Events** | **Severity** | **Related to Study** |
| --- | --- | --- | --- |
| **Control** | Rash | Mild | Assessed as unlikely related to control material. The condition pre-existed study commencement and recurred during the study. |
|  | Diarrhea | Mild | Possibly related to control material |
|  | Bloating | Mild | Possibly related to control material |
| **Treatment** | None reported |  |  |

**Table S2: Data and statistical model output.**

Supplementary file “Table S2 Data and Statistics.xlsx”

**Table S3: Probiotic composition**

| Genus | species |
| --- | --- |
| *Bifidobacterium* | *breve* |
| *Bifidobacterium* | *lactis* |
| *Streptococcus* | *thermophilus* |
| *Lactobacillus* | *plantarum* |
| *Lactobacillus* | *paracasei* |
| *Lactobacillus* | *delbrueckii* |
| *Lactobacillus* | *acidophilus* |

***Supplementary Figures***

**Figure S1: CONSORT report chart**.


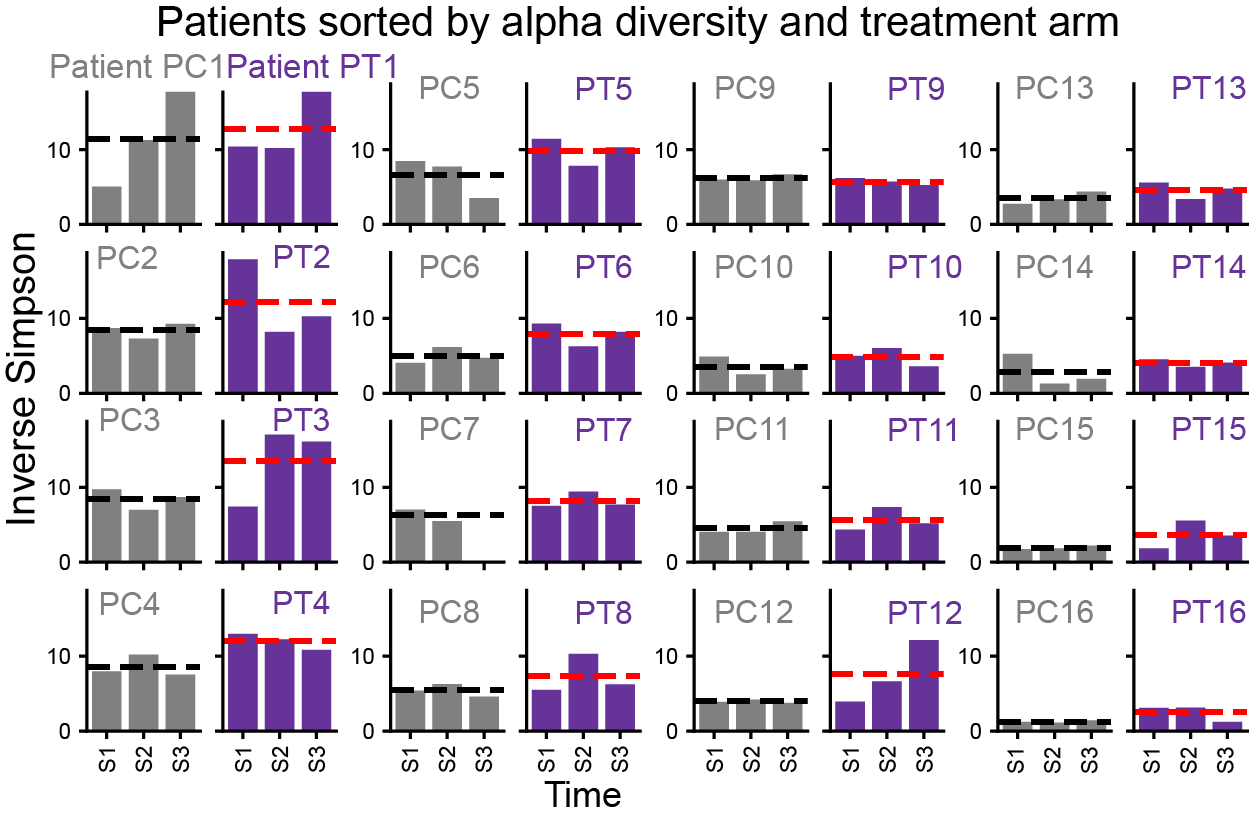


**Figure S2: Higher alpha diversity observed across individual patients during primary endpoints (S1-S3)**. Inverse Simpson alpha diversity in 32 patients (Control: PC, Treated: PT) at the end of an antibiotic course across samples S1, S2, S3; patients sorted by trial arm and average diversity in descending order from top left to bottom right. Dashed lines indicate control (grey) and treated (red) patient averages.

**
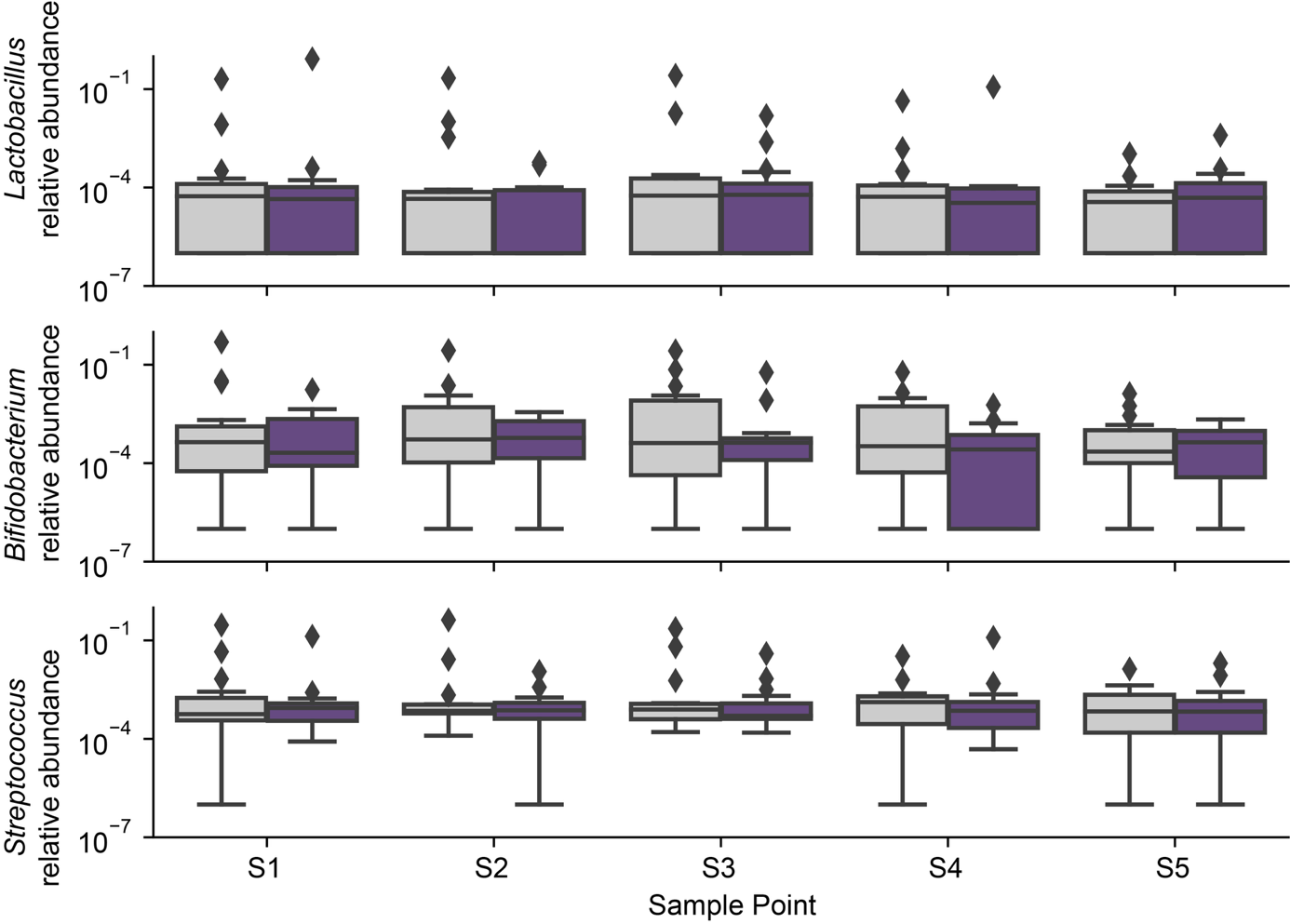
**

**Figure S3: Relative abundances of probiotic genera**. Relative genus abundances shown for samples from control (grey) and treated (purple) patients; differences were non-significant at each time point between treatment arms (Wilcoxon rank sum tests).


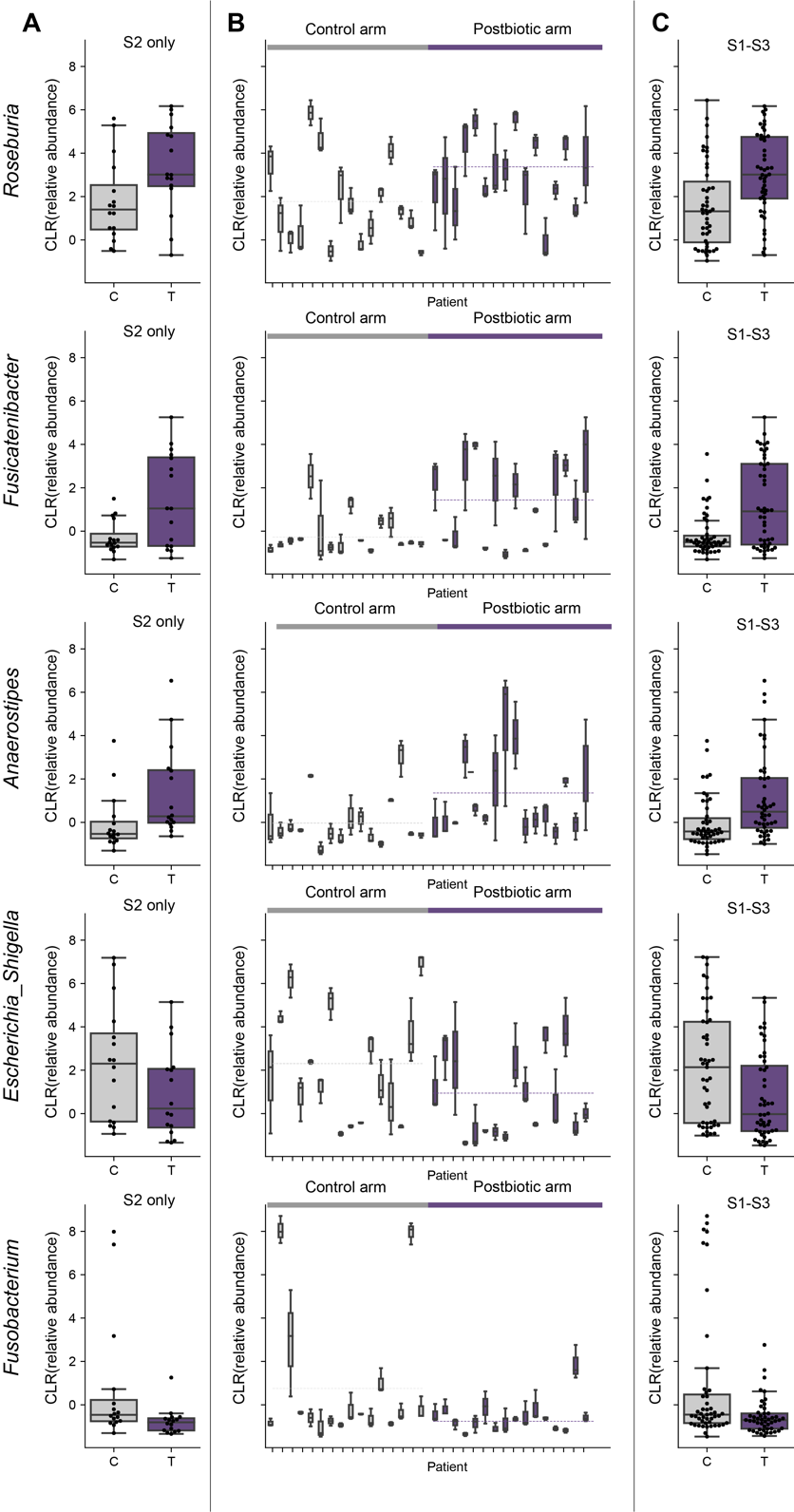


**Figure S4: CLR transformed relative abundances of five most strongly associated genera across patients and time.** Genera are organized in rows. **A**) CLR transformed relative abundances by treatment arm at timepoint S2 alone, **B)** for each patient in timepoints S1, S2, and S3 (dashed lines indicate medians per treatment arm), **C)** by treatment arm in timepoints S1, S2, and S3.

**
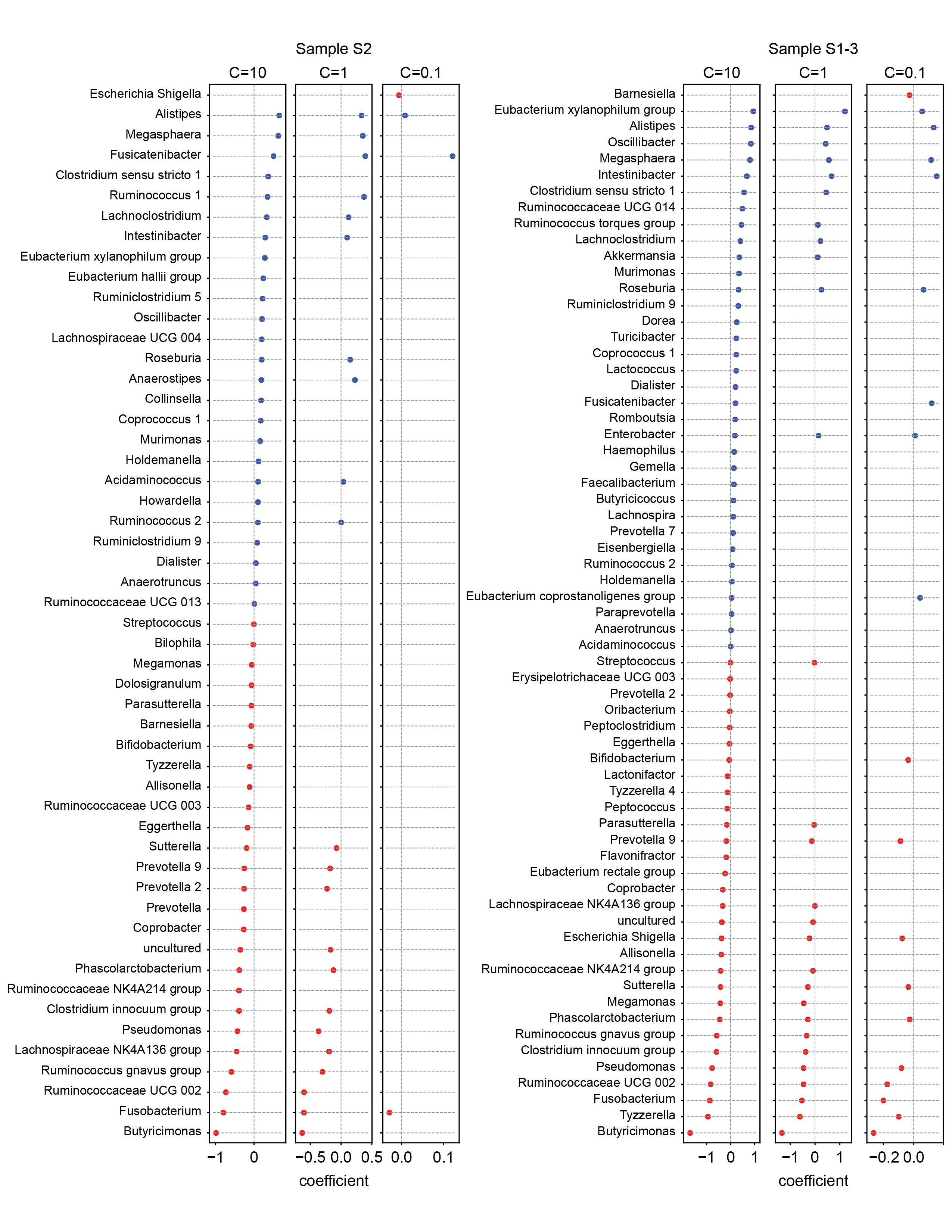
**

**Figure S5: Multivariate logistic regression with varying regularization strengths (C) confirm univariate results.** Coefficient estimates from L1-penalized logistic regressions on performed on CLR-transformed genus relative abundances in Sample S2 (**A**), or samples S1-S3 (**B**), at different regularization strengths up until all coefficients were set to zero (C: inverse regularization strength).

**
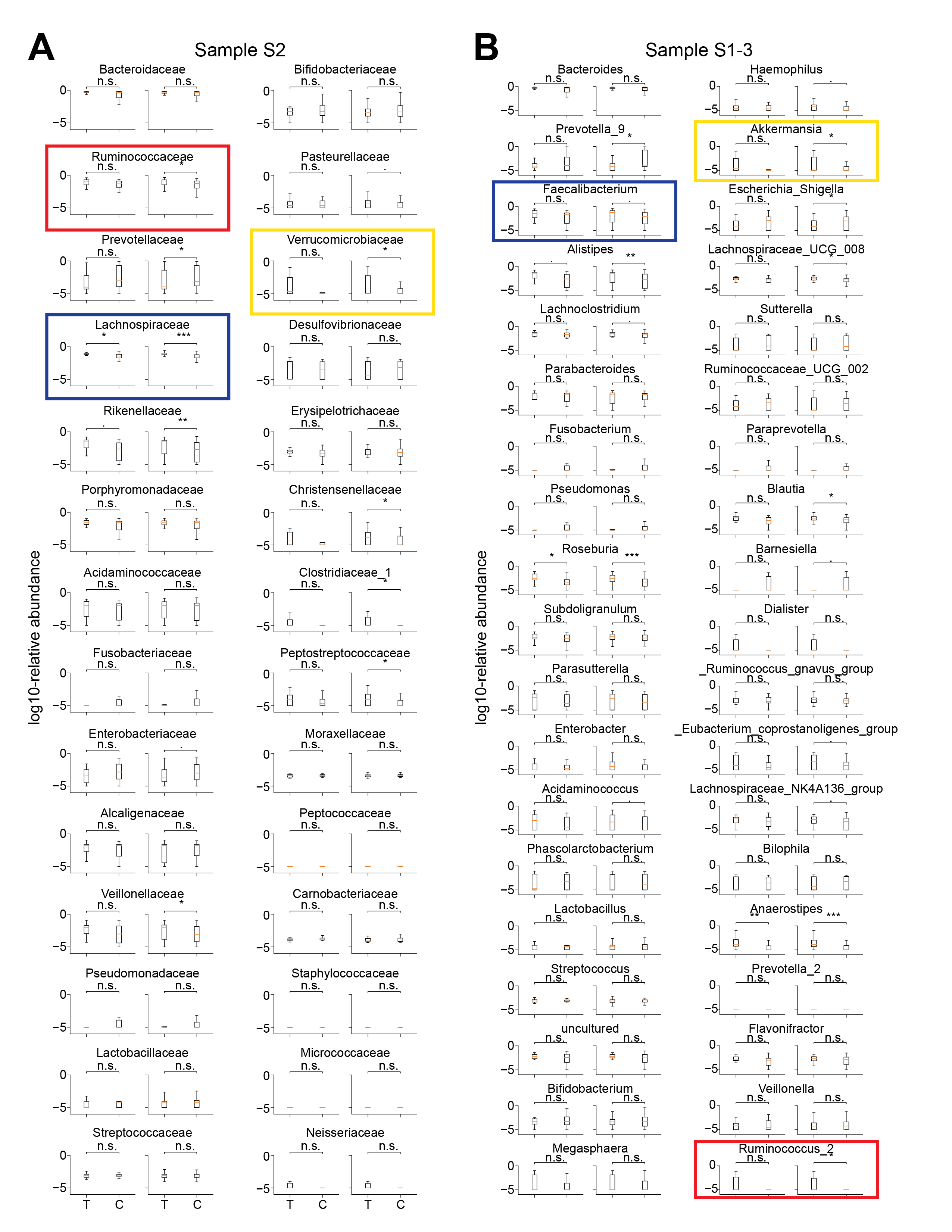
**

**Figure S6: Relative abundance of bacterial families and genera**, sorted by average abundance. Log-10 transformed relative family abundances in sample S2 (**A**) and genus (**B**) abundances pooled over samples S1-S3 in treated (T) and control (C) patients. Boxes highlight select taxa discussed as modulating cancer immunotherapies^15,32,33^. ***: p<0.001, **: p<0.01, *: p<0.05, .: p<0.1, n.s.: p>0.1; non-parametric Wilcoxon rank sums test not corrected for multiple hypotheses.

**Figure S7: Multivariate L-1 regularized logistic regression of late samples.** Coefficient estimates from L1-penalized logistic regressions on performed on CLR-transformed genus relative abundances in Sample S4 and S5 (**A**), or samples S5 alone (**B**) (C: inverse regularization strength).


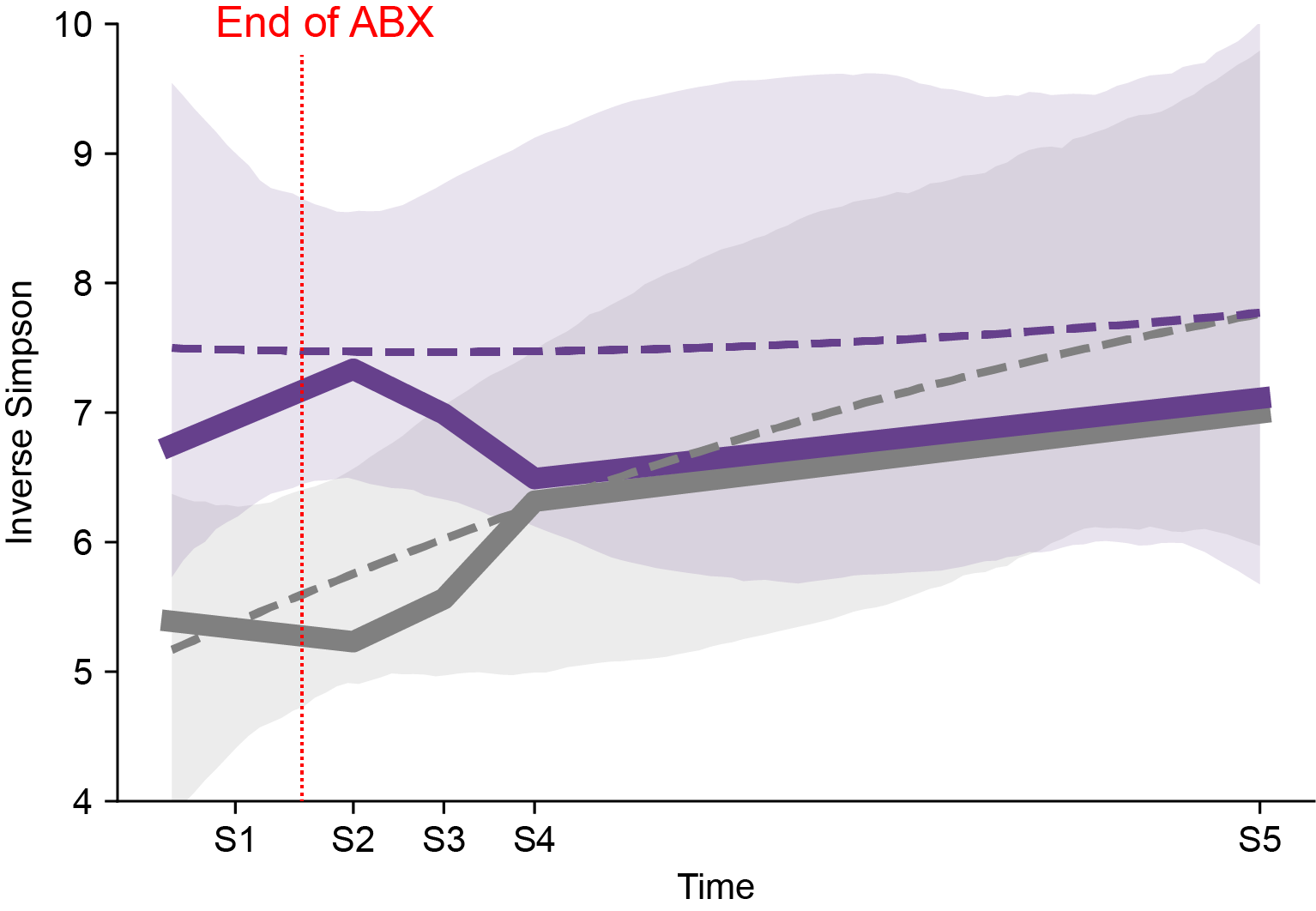


**Figure S8: Bacterial alpha diversity is increased at the end of an antibiotic course in patients who received a postbiotic during their treatment and becomes similar ten days after finishing the antibiotic.** Thick lines show a locally weighted scatterplot smoothing curve for treated (purple) and control (grey) patients’ bacterial alpha diversity across five longitudinally collected samples; dashed lines and shaded regions show mean and confidence intervals of a second-order line fit with time in days as predictor of alpha diversity.
